# Delayed Presentation of Duodenal Atresia in a Male With Trisomy 21

**DOI:** 10.7759/cureus.21700

**Published:** 2022-01-28

**Authors:** Annalisa G Sega, Teerin Meckmongkol, Tamarah Westmoreland

**Affiliations:** 1 Medicine, University of Central Florida College of Medicine, Orlando, USA; 2 Pediatric Surgery, Nemours Children's Hospital, Orlando, USA; 3 Pediatric Surgery, University of Central Florida College of Medicine, Orlando, USA

**Keywords:** intestinal atresia, obstruction, emesis, congenital malformation, embryogenesis

## Abstract

The duodenum is the secondmost common site of congenital intestinal obstruction. There are three types of congenital duodenal atresia according to the severity of obstruction. Duodenal atresia is thought to develop due to the failure of recanalization of the gut lumen during embryonic development. This congenital abnormality usually presents in utero or shortly after birth with signs of intestinal obstruction. However, rare cases can present later in life.

In this case report, we will discuss a two-year-old male with trisomy 21 who presented with intractable vomiting and failure-to-thrive. He did not have the classic clinical or diagnostic signs of duodenal atresia, but on exploratory laparotomy, he was found to have severe duodenal stenosis. Diamond-shaped duodenoduodenostomy was performed to bypass the stenosed intestine. The patient recovered well from surgery and was able to tolerate a soft mechanical diet without vomiting one week postoperatively. This case exhibits a particularly delayed and atypical presentation of duodenal stenosis. Yet, it is imperative to recognize this presentation from an educational and clinical standpoint for surgical intervention.

## Introduction

Duodenal atresia is a congenital intestinal obstruction that can cause bilious or nonbilious vomiting usually within the first days to weeks of life, typically following oral feeding. It is often associated with in-utero polyhydramnios and is one of the most common causes of fetal bowel obstruction. There are three types of duodenal atresia, ranging from least to most severe. Type 1 duodenal atresia is defined as duodenal webs, where mucous membranes can cover part of the gut lumen causing a partial obstruction. Patients may not have gastric distention but may have episodic vomiting or feeding intolerance. In type 2 duodenal atresia, duodenal stenosis, the atretic ends of the duodenum are connected by a fibrous cord. This cord may or may not be patent, allowing some gastric material to pass through. In type 3 duodenal atresia, the atretic segments are completely separated, a true atresia [1,2]. Infants with this type of duodenal atresia will have gastric distention and vomiting that are often, but not always, bilious.

The bilious content of the emesis depends on the location of the atresia with relationship to the ampulla of Vater. If the stenosis is before the ampulla, vomiting is nonbilious; if it is past the ampulla, the vomiting will be bilious. Some infants with duodenal atresia may pass meconium. Patients can also have poor feeding, failure-to-thrive, be small for gestational age, or premature. Excessive vomiting can cause hypokalemic hypochloremic metabolic alkalosis. Patients can have symptoms of abdominal distension and absent bowel movements. Duodenal atresia can be diagnosed prenatally on prenatal ultrasound, as well as a few days after birth by abdominal plain film or upper gastrointestinal (GI) imaging. The plain film will often show a “double bubble” sign, indicating air in the stomach and duodenum proximal to obstruction with absent distal gas [3].

The etiology of duodenal atresia is thought to come from a failure of recanalization during embryonic development. The duodenum is derived partly from the embryonic foregut and partly from the midgut [4]. During development, the endoderm proliferates and occludes the gut lumen. Vacuoles then recanalize the lumen to restore patency during eight to 10 weeks gestation. Duodenal atresia occurs due to the failure of this process; complete failure leads to type 3, and partial failure leads to type 1 or 2 [2].

It is important to note the differences in etiology between duodenal atresia and intestinal atresia. While duodenal atresia is hypothesized to occur from a failure of recanalization, cases of intestinal atresia, including jejunal, ileal, and colonic, are thought to occur due to vascular injury. A vascular disruption during development leads to necrosis of a segment of fetal tissue, which is then resorbed, leaving blind proximal and distal ends of the intestine [5].

It is also important to differentiate the clinical presentations of intestinal obstruction. While most cases of duodenal atresia present early with abdominal distention and vomiting, many other abnormalities can present similarly. For example, congenital duodenal atresia due to failure of recanalization presents similar to duodenal stenosis due to annular pancreas, as can pyloric stenosis. Pyloric stenosis will present with nonbilious vomiting because the obstruction occurs before the ampulla of Vater [6]. However, the most important condition to eliminate from the differential diagnosis for an infant or child with feeding intolerance, abdominal pain, distension, and vomiting is malrotation with midgut volvulus. Malrotation with midgut volvulus is a surgical emergency due to the potential for necrosis and perforation [7]. This can be ruled out with an upper GI study.

Duodenal atresia is often associated with other congenital abnormalities. Up to 30% of duodenal atresia cases are associated with abnormal karyotypes [8]. Specifically, duodenal atresia is 265x higher in infants with trisomy 21 than those without [8]. In infants with duodenal atresia without an abnormal karyotype, further investigation should still be pursued, especially before surgical intervention. Most importantly, patients should have an echocardiogram to evaluate for congenital heart disease such as endocardial cushion defects as up to 50% of patients with duodenal atresia will have cardiac defects [9]. An abdominal ultrasound can also be done to evaluate for biliary atresia and esophageal atresia. Other anomalies such as genitourinary and skeletal anomalies can also be seen.

## Case presentation

A two-year-old male was admitted for concerns of duodenal atresia on the upper GI scan due to repeated bouts of emesis and a history of trisomy 21. His mother stated that the patient has always had “reflux” and would vomit after feeds. The patient also suffered from oropharyngeal dysphagia. Over the last few months, he has been unable to tolerate enough calories to gain weight, and his pediatrician was concerned that he was not growing appropriately. The patient was referred to the gastroenterology service, and an upper GI study was done, which showed abnormality findings suspicious of duodenal atresia. It was not previously detected on other imaging studies, including prenatal ultrasounds and postnatal plain films.

The patient was prenatally diagnosed with trisomy 21 via chorionic villus sampling. He had a history of tetralogy of Fallot, bladder diverticulum, and hypothyroidism. He had surgical repairs of his tetralogy of Fallot and bladder diverticulum at another hospital from 2019 to 2020. He also had tympanostomy tubes placed, and his adenoids were removed in 2020. His mother expressed no ongoing concerns with those procedures or conditions. He also presented with stage 2 chronic kidney disease and was being followed by the nephrology service. He was taking cyproheptadine and levothyroxine daily. The patient was the only child of both his parents. He lived at home and did not attend daycare. Family history was significant for diabetes mellitus type 2, thyroid disease, and hypertension in his maternal grandmother and hypertension in his maternal grandfather. Parents reported no significant medical conditions.

On admission, vital signs were within normal limits. On examination, the patient was well-appearing and was not in distress. Lungs were clear to auscultation, and heart sounds revealed S1 and S2 with no murmurs. The abdomen was slightly distended but nontender, with no rebound tenderness or guarding. Extremities were well perfused with a capillary refill of 3 seconds. It is important to note that this patient did not have a tense or distended abdomen and had no acute abdominal pain; there was also no current emesis.

**Figure 1:**
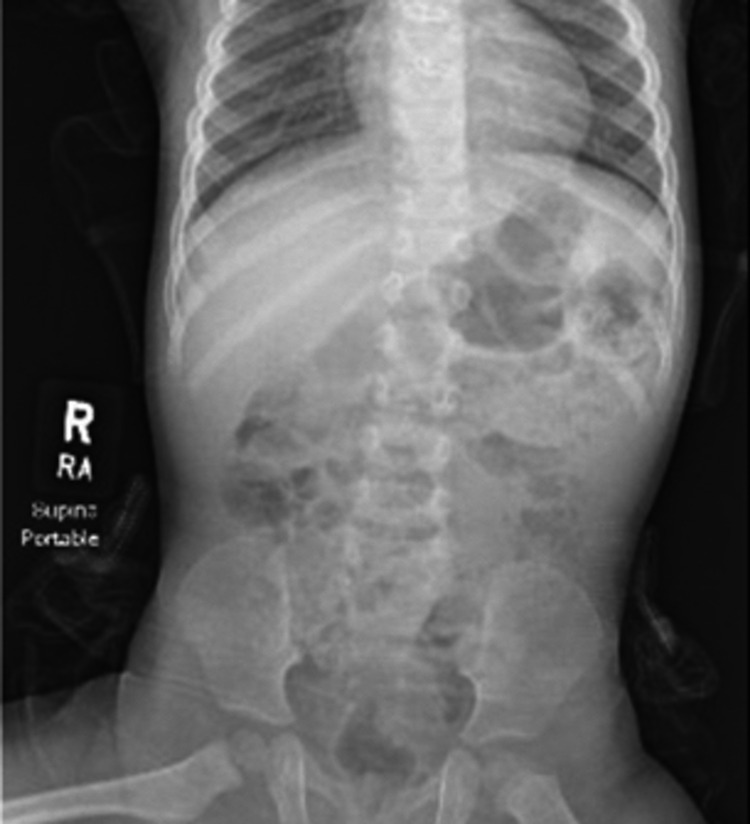
Single-view abdominal plain film showing distal bowel gas Single-view abdominal plain film showing normal caliber gas and stool in the proximal and distal bowel, with no evidence of obstruction. There is mild-to-moderate gaseous distention, without the classic double "bubble sign" of one gastric air bubble and one proximal duodenal air bubble, that would suggest duodenal atresia.

The Upper GI contrast study showed a collection of contrast material in a dilated cone-shaped duodenum, suggesting duodenal web or stenosis. The abdominal plain film showed gaseous distension with gas and stool in the distal bowel, suggesting a lack of complete obstruction (Figure 1). It is important to note that this patient did not have the classic sign of a “double bubble” on the abdominal plain film.

The patient was managed according to the procedures discussed below, and he tolerated the surgery very well. During surgery, the patient was noted to have numerous Ladd’s bands, which were removed. He had a portion of the duodenum that was significantly stenosed and much smaller in diameter than the proximal portion. A red rubber catheter was passed through the stenosed portion to ensure webs were present. Past the stenosed area, the jejunum opened back out to normal diameter. Saline was passed from the distal longitudinal incision through the jejunum to ensure no downstream stenosis. Following approximation, a Dobbhoff feeding tube was passed from the right nare through the stomach and the new anastomosis to ensure patency. The tube was then left in the jejunum for nasojejunal (NJ) feeds postoperatively. A 10Fr Blake drain was placed inferior to the anastomosis site and secured to the skin with sutures. The patient was extubated and awakened from anesthesia without complications. He remained nothing by mouth (NPO) for seven days, with intravenous (IV) fluids and nasogastric (NG) suction. He began NJ tube feeds after three days, and he began taking oral feeds after seven days following a repeat upper GI follow-through study showing patency of the anastomosis without leaks.

Follow-up for this patient was done in the general surgery clinic two weeks after the surgery. At that time, the patient was tolerating per os (PO) intake on a soft mechanical diet without complications and with a decrease in vomiting. His incision was healing well with no postoperative complications. Long-term follow-up for this patient was scheduled for the future.

## Discussion

The timeline, in this case, presents much differently than most congenital intestinal obstruction cases. Duodenal atresia typically presents prenatally or in the early postnatal period. In this case, the patient presented very late, at two years of age, presumably because his duodenal atresia did not cause complete obstruction. Instead, it caused a partial obstruction with which some digestive material was still able to pass. As such, he was able to eat and process some nutrients but not enough to maintain adequate nutrition for growth. He was also unable to avoid symptoms: He suffered from intractable vomiting and reflux. Due to his medical history of trisomy 21, it was hypothesized that he had type 1 or 2 duodenal atresia, duodenal webs, or stenosis. Imaging studies and surgical exploration deduced he suffered from type 2 (stenosis) of the duodenum. Even though he had survived this long with a stenosed duodenum, he was not eating or growing well, so this issue required surgical correction.

The initial management for duodenal atresia is similar to bowel obstruction: NPO, nasogastric (NG) suction, and IV fluids. These steps are to stabilize the patient before surgery. The NPO order is to ensure the bowel is ready for the operation and to prevent aspiration with anesthesia. Because many of these patients have a history of vomiting, the IV fluids are to ensure that they are well hydrated and to begin to correct electrolyte abnormalities. The procedure to correct the defect is an open duodenoduodenostomy; the two atretic ends of the duodenum are connected back together. This is frequently accomplished through a “double-diamond” procedure. First, a transverse incision is made on the proximal portion of the duodenum, and a longitudinal incision is made on the distal portion. The midpoints of the transverse incisions are then approximated to the proximal and distal apexes of the longitudinal incision, resulting in a diamond-shaped anastomosis [10,11]. The benefits of this operation are a shorter time to oral feeding as well as a shorter postoperative hospital course.

A newer treatment option that is emerging in the literature for duodenal webs is the use of endoscopic balloon dilation [12,13]. Although this procedure was not used for this patient with type 2 duodenal stenosis, in patients with tight duodenal stenosis caused by webs with fenestration (type one), endoscopic dilation can be used, and the residual septum can be removed by electrocautery if needed. This procedure has the benefits of shorter operation time, less scarring, and earlier recovery.

The survival of patients with duodenal atresia is 95% [4]. Most mortality occurs in infants with medical conditions such as prematurity or respiratory distress syndrome, other associated anomalies, or complications such as short gut syndrome. Risk factors for death include complex cardiac defects, sepsis, and low birth weight. Common postoperative complications include congestive heart failure, ileus, gastroesophageal reflux disease, anastomosis obstruction, and postoperative wound infection.

## Conclusions

Duodenal atresia can present prenatally, during the neonatal period, and, rarely, in a young child. Diagnosis is based on clinical and imaging findings. This presentation is an important educational and clinical case because it is an unlikely timeline for this disease process. Two years of age is a late presentation of duodenal atresia, even with atypical or less severe symptoms. It is imperative to not miss this diagnosis because the repair is primarily surgical.
